# Supplementary material for: Complete remission of diabetes with a transient HDAC inhibitor and insulin in streptozotocin mice
Source: Commun Biol. 2023 Jun 13;6:637. doi: 10.1038/s42003-023-05010-x (PMC10264456; doi:10.1038/s42003-023-05010-x)
Supplement: Supplementary file 2 — Supplemental Information [file 42003_2023_5010_MOESM2_ESM.pdf]

Supplementary Figure 1

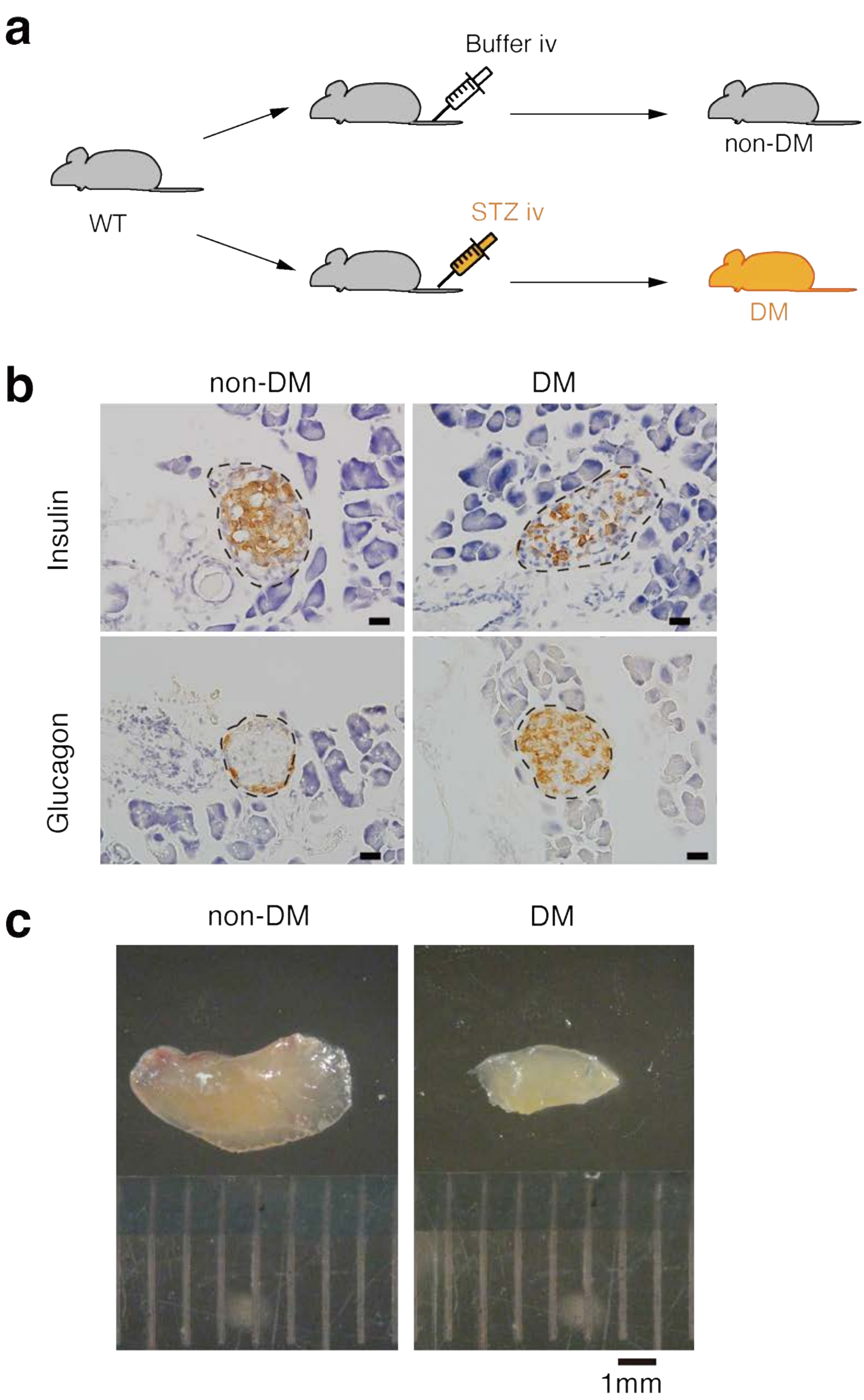

Supplementary Fig. 1. (a) Citrate buffer (pH 4.5) or STZ (150 mg/kg) in citrate buffer (pH 4.5) was injected into the tail veins of male C57BL6/J mice to induce nondiabetes (non-DM) or diabetes (STZDM), and all mice were sacrificed at 12 weeks. Gray mice indicate non-DM, and orange mice indicate DM mice. White syringe shows control buffer injection whereas orange indicates STZ injection. (b) Immunohistochemistry for insulin (upper panels) and glucagon (lower panels) are shown. Positive signals are shown in brown. (c) Pictures of the thymus mass in DM (n=1) and non-DM mice (n=1).

Supplementary Figure 2

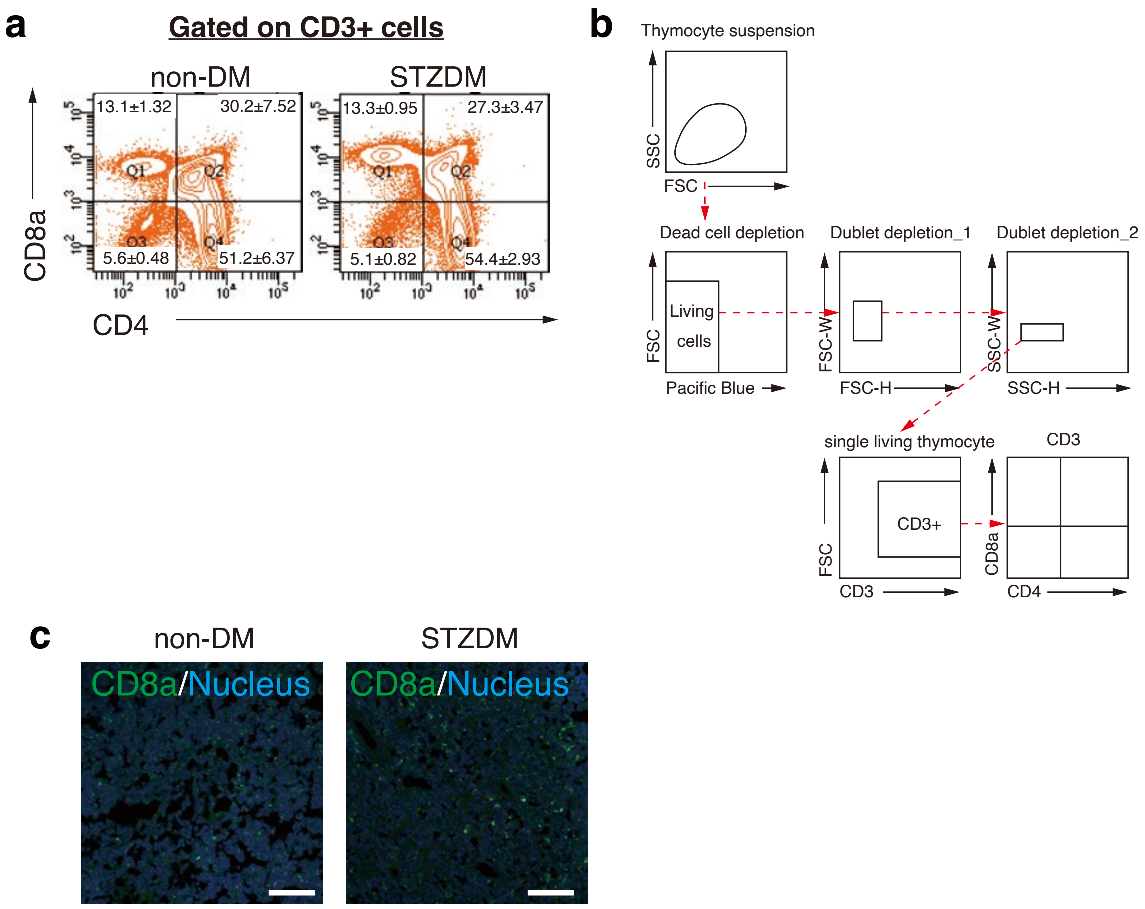

Supplementary Fig. 2. The effect of streptozotocin on T cells in the thymus of diabetic and non-diabetic mice. (a) Dot plot diagrams showed that the fractions of CD4+ (bottom right quadrant), CD8+ (top left quadrant), and both positive (top right quadrant) cells in total CD3+ cells in control non-diabetic mice (n=4) are also similar to those in STZ-induced diabetic mice (n=4). A portion of unstained suspended cells was stained with an isotype control of each fluorescent antibody to determine gating. Data are indicated as means ± SE. (b) Gating strategy for FACS analysis of thymocyte suspension from non-DM and STZDM. (c) immunohistochemistry showed that the number of CD8a in the thymus of non-diabetic mice were not different from those in diabetic mice.

Supplementary Figure 3

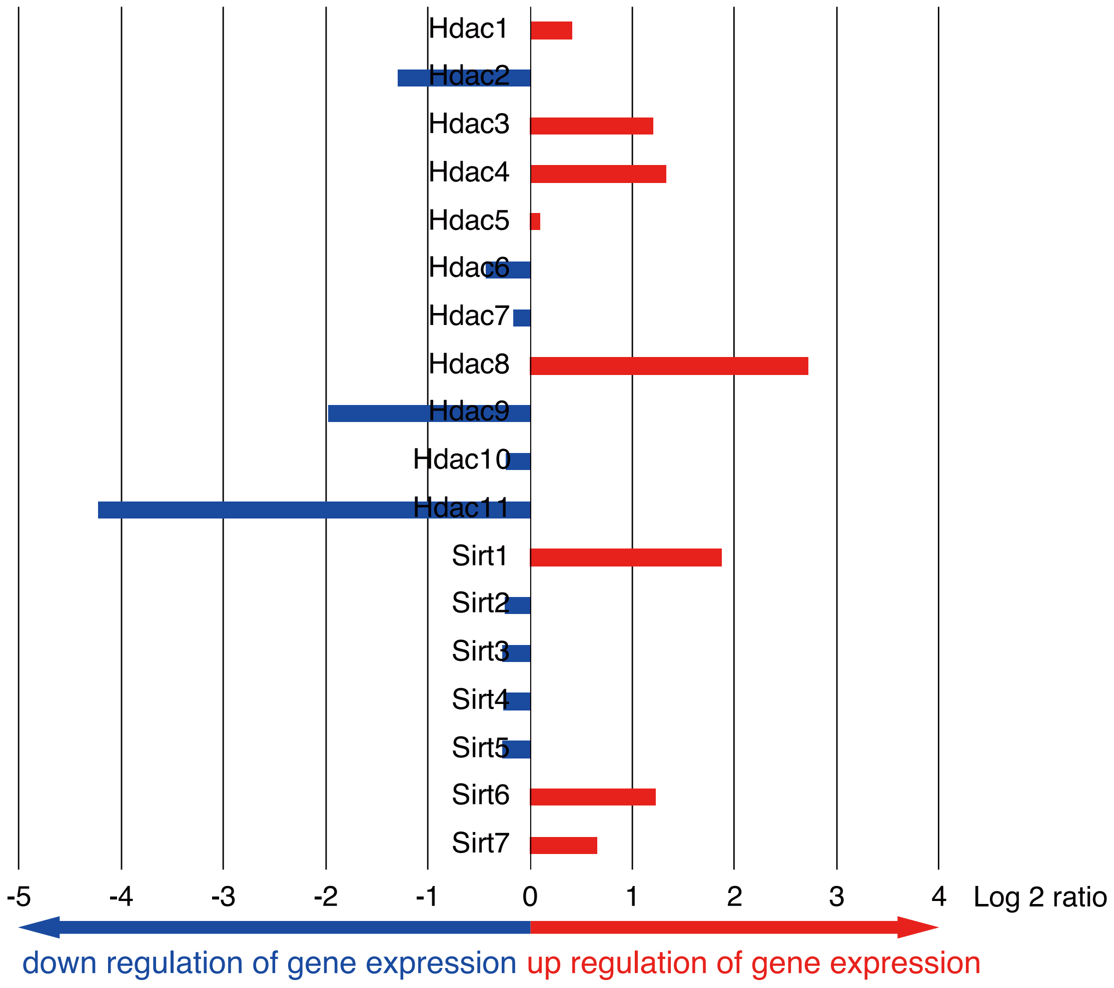

Supplementary Fig. 3. Altered HDAC expression in LSK cells in the bone marrow of diabetic mice. The mRNA expression levels for several types of HDACs examined by microarray analysis are shown (Gene Expression Omnibus (GEO) accession No. GSE224690).

Supplementary Figure 4

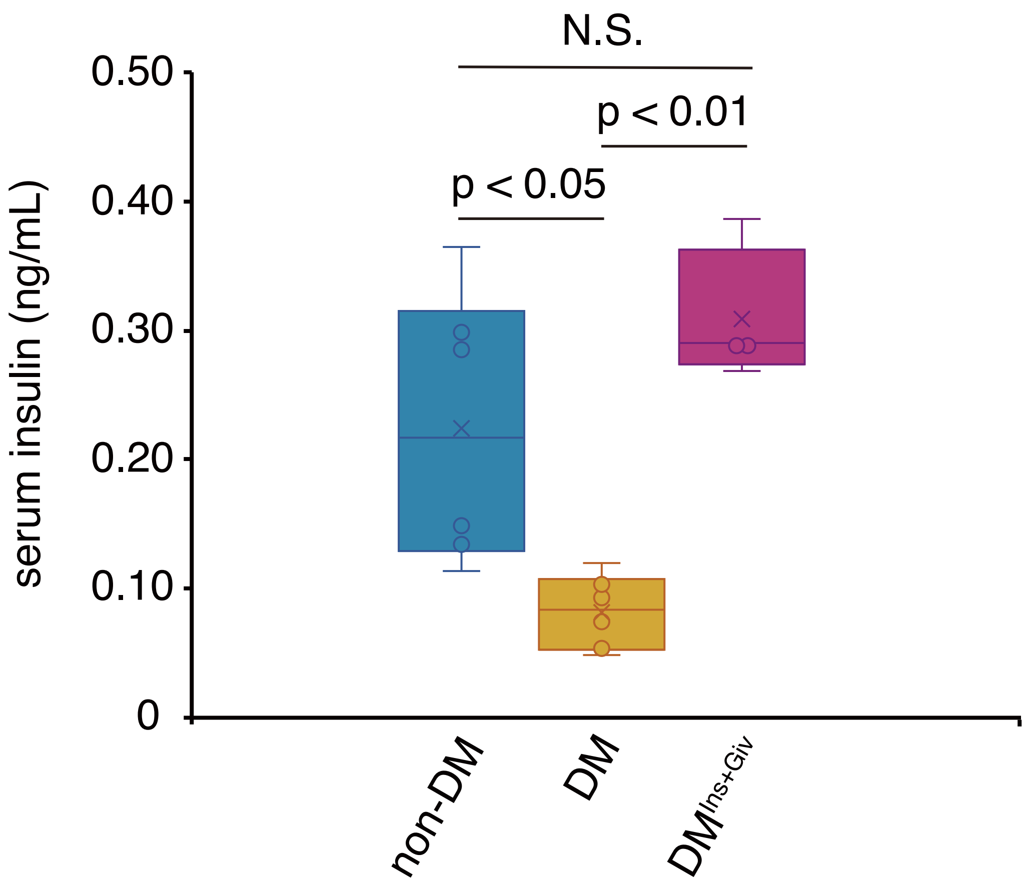

Supplementary Fig. 4. The box plot of combination therapy restored serum insulin concentrations at the end of the study in diabetic mice (non-DM (n=6), DM (n=6), $DM^{Ins+Giv}$ (n=4)).

Supplementary Figure 5

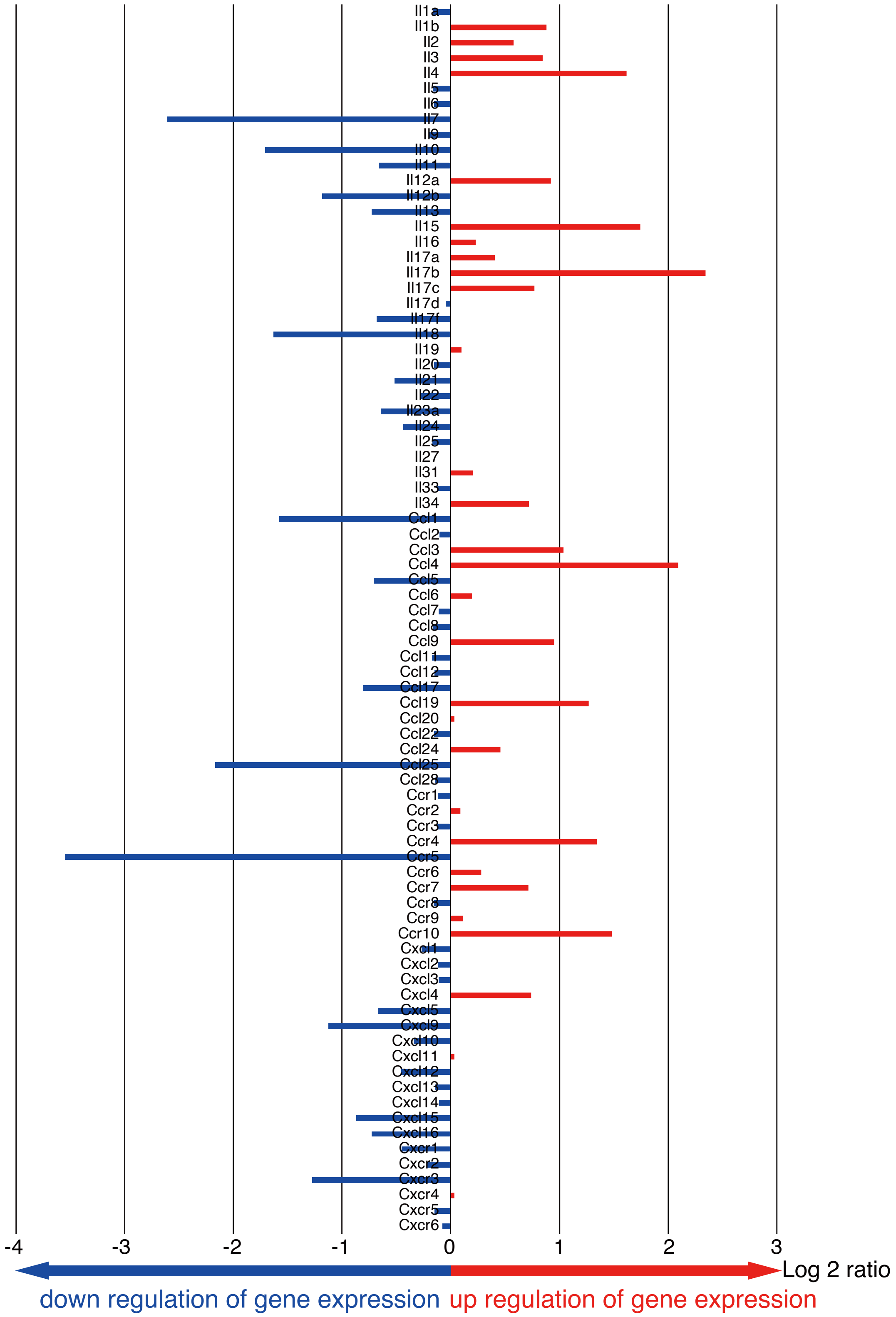

Supplementary Fig. 5. Altered chemokine expression in LSK cells in the bone marrow of diabetic mice (Gene Expression Omnibus (GEO) accession No. GSE224690).

Supplementary Figure 6

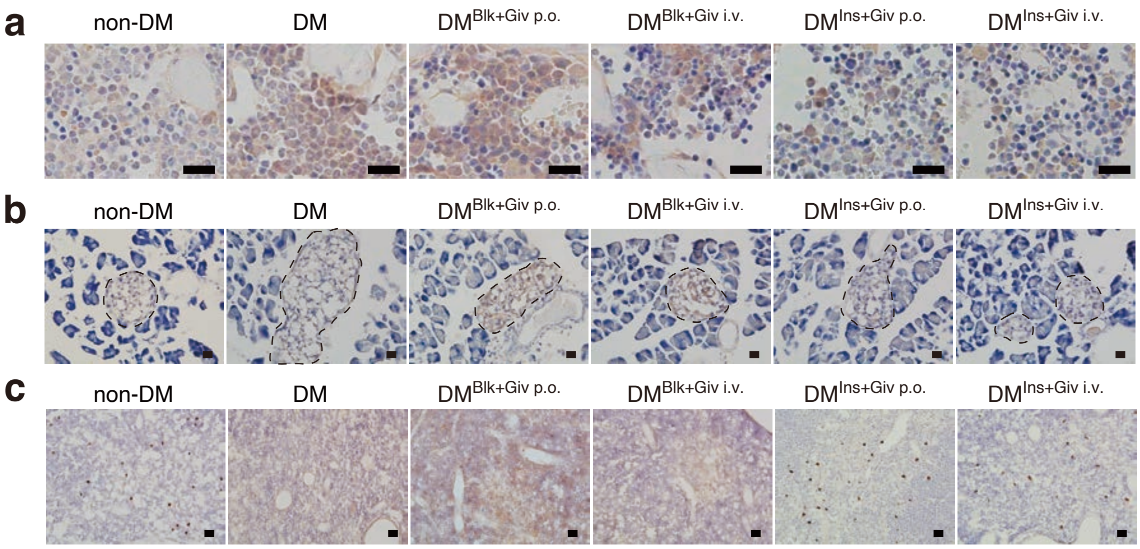

Supplementary Fig. 6. Combination therapy reduces TNF-α expression in the BM, pancreas and thymus and recovers thymus mass in diabetic mice.

Immunohistochemical analysis of TNF-α in the BM (a), pancreas (b) and thymus (c). Scale bars =20 μm.
