# Supplementary material for: Complete remission of diabetes with a transient HDAC inhibitor and insulin in streptozotocin mice
Source: Commun Biol. 2023 Jun 13;6:637. doi: 10.1038/s42003-023-05010-x (PMC10264456; doi:10.1038/s42003-023-05010-x)
Supplement: Supplementary file 3 — Description of Additional Supplementary Files [file 42003_2023_5010_MOESM3_ESM.pdf]

Description of Additional Supplementary Files

File name: Supplementary Data 1

Description: Original data for the graphs used in the paper.
