# Supplementary material for: Complete remission of diabetes with a transient HDAC inhibitor and insulin in streptozotocin mice
Source: Commun Biol. 2023 Jun 13;6:637. doi: 10.1038/s42003-023-05010-x (PMC10264456; doi:10.1038/s42003-023-05010-x)
Supplement: Supplementary file 5 — Reporting Summary [file 42003_2023_5010_MOESM5_ESM.pdf]

nature portfolio

Corresponding author(s): Takahiko Nakagawa

Last updated by author(s): May 19, 2023

Reporting Summary

Nature Portfolio wishes to improve the reproducibility of the work that we publish. This form provides structure for consistency and transparency in reporting. For further information on Nature Portfolio policies, see our Editorial Policies and the Editorial Policy Checklist.

Statistics

For all statistical analyses, confirm that the following items are present in the figure legend, table legend, main text, or Methods section.

n/a	Confirmed	
☐	☒	The exact sample size (n) for each experimental group/condition, given as a discrete number and unit of measurement
☐	☒	A statement on whether measurements were taken from distinct samples or whether the same sample was measured repeatedly
☐	☒	The statistical test(s) used AND whether they are one- or two-sided<br>*Only common tests should be described solely by name; describe more complex techniques in the Methods section.*
☒	☐	A description of all covariates tested
☒	☐	A description of any assumptions or corrections, such as tests of normality and adjustment for multiple comparisons
☒	☐	A full description of the statistical parameters including central tendency (e.g. means) or other basic estimates (e.g. regression coefficient) AND variation (e.g. standard deviation) or associated estimates of uncertainty (e.g. confidence intervals)
☐	☒	For null hypothesis testing, the test statistic (e.g. F, t, r) with confidence intervals, effect sizes, degrees of freedom and P value noted<br>*Give P values as exact values whenever suitable.*
☒	☐	For Bayesian analysis, information on the choice of priors and Markov chain Monte Carlo settings
☒	☐	For hierarchical and complex designs, identification of the appropriate level for tests and full reporting of outcomes
☒	☐	Estimates of effect sizes (e.g. Cohen's d, Pearson's r), indicating how they were calculated

Our web collection on statistics for biologists contains articles on many of the points above.

Software and code

Policy information about availability of computer code

Data collection: C1si confocal microscope (Nikon), TCS SP8 X confocal microscope (Leica), FreeStyle (Nipro),

Data analysis: EZ-C1s software (Nikon), LAS X small (Leica), Illustrator 2022 (Adobe), Image J 1.53(NIH), Excel (Microsoft)

For manuscripts utilizing custom algorithms or software that are central to the research but not yet described in published literature, software must be made available to editors and reviewers. We strongly encourage code deposition in a community repository (e.g. GitHub). See the Nature Portfolio guidelines for submitting code & software for further information.

Data

Policy information about availability of data

All manuscripts must include a data availability statement. This statement should provide the following information, where applicable:

- Accession codes, unique identifiers, or web links for publicly available datasets
- A description of any restrictions on data availability
- For clinical datasets or third party data, please ensure that the statement adheres to our policy

we got accession number from GEO.
(Accession number GSE224690)

Research involving human participants, their data, or biological material

Policy information about studies with human participants or human data. See also policy information about sex, gender (identity/presentation), and sexual orientation and race, ethnicity and racism.

Reporting on sex and gender	We did not perform human research.
Reporting on race, ethnicity, or other socially relevant groupings	We did not perform human research.
Population characteristics	We did not perform human research.
Recruitment	We did not perform human research.
Ethics oversight	We did not perform human research.

Note that full information on the approval of the study protocol must also be provided in the manuscript.

Field-specific reporting

Please select the one below that is the best fit for your research. If you are not sure, read the appropriate sections before making your selection.

☒ Life sciences ☐ Behavioural & social sciences ☐ Ecological, evolutionary & environmental sciences

For a reference copy of the document with all sections, see nature.com/documents/nr-reporting-summary-flat.pdf

Life sciences study design

All studies must disclose on these points even when the disclosure is negative.

Sample size	We have used animals for our experiments. Sample sizes were set as minimum as possible to detect the statistical significance in each experiment.
Data exclusions	Before the collection of the data, we set the design that we can obtain fair decision.<br>For that purposes, we exclude animals that shows abnormal condition such as extraordinary low body weight or healthy problems before the data sampling.
Replication	We did not do replicated experiment in all studies.
Randomization	We did randomization of all animals and samples to adjust the study design.
Blinding	Data from each animal or each sample were obtained under blindness and with no experimenter's bias.

Reporting for specific materials, systems and methods

We require information from authors about some types of materials, experimental systems and methods used in many studies. Here, indicate whether each material, system or method listed is relevant to your study. If you are not sure if a list item applies to your research, read the appropriate section before selecting a response.

Materials & experimental systems

n/a	Involved in the study	
☐	☒	Antibodies
☒	☐	Eukaryotic cell lines
☒	☐	Palaeontology and archaeology
☐	☒	Animals and other organisms
☒	☐	Clinical data
☒	☐	Dual use research of concern
☒	☐	Plants

Methods

n/a	Involved in the study	
☒	☐	ChIP-seq
☐	☒	Flow cytometry
☒	☐	MRI-based neuroimaging

Antibodies

Antibodies used	Rabbit anti-insulin monoclonal antibody (clone C27C9, Cell Signaling Technologies, Danvers, MA USA, #3014, Lot: 8, 1:800)<br>rabbit anti-glucagon polyclonal antibody (Cell Signaling Technologies, #2760, Lot: 2, 1:200)

rabbit anti-TNF-α polyclonal antibody (Abcam, Cambridge, UK, #ab6671, 1:100)
Vcam-1 Rabbit monoclonal antibody (mouse specific) (clone D8U5V, Cell signaling Technologies, #39036, Lot: 1, 1:400)
CSPG4, NF2 Rabbit Polyclonal antibody (Proteintech, #55027-1-AP, 1:100)
Anti-Von Willebrand Factor polyclonal antibody (Abcam, #ab11713, Lot:GR3197920-3, 1:100)
Goat anti-Rabbit IgG (H+L) Cross-Adsorbed Secondary Antibody, Alexa Fluor 488 (Thermo Fisher Scientific Inc. Waltham, MA, USA #A-11008, Lot: 2521157, 1:1000)
Donkey anti-Sheep IgG (H+L) Cross-Adsorbed Secondary Antibody, Alexa Fluor 488 (Thermo Fisher Scientific Inc. #A-11015, Lot: 1900213, 1:1000)
Donkey anti-Rabbit IgG (H+L) Highly Cross-Adsorbed Secondary Antibody, Alexa Fluor 555 (Thermo Fisher Scientific Inc. #A-31572, Lot: 2088692, 1:1000)
Purified anti-mouse CD16/32 antibody (Clone 93, Biolegend, #101301, 0.5μg/100μl)
PE/Cyanine7 anti-mouse CD3 antibody (Clone 17A2, Biolegend, #100219, 0.1μg/100μl)
FITC anti-mouse CD4 (Clone GK1.5, Biolegend, #100405, 0.25μg/100μl)
APC anti-mouse CD8a (Clone 53-6.7, Biolegend, #100711, 0.1μg/100μl)

Validation

We decided optimal concentration and reaction time of all antibodies before our study started.

Animals and other research organisms

Policy information about studies involving animals; ARRIVE guidelines recommended for reporting animal research, and Sex and Gender in Research

Laboratory animals

C57BL/6J mouse (Japan SLC),
C57BL/6-Tg (UBC-GFP) 30Scha/J mice (The Jackson laboratory, Stock #004353),
B6.Cg-Gt(ROSA)26Sortm9(CAG-tdTomato)Hze/J (The Jackson laboratory, Stock #007909),
Ayu1 promoter-driven Cre recombinase-expressing mice (Niwa H et.al, 1993)

Wild animals

No.

Reporting on sex

male

Field-collected samples

No.

Ethics oversight

Our animal studies were approved by the Animal Care Committees of Shiga University of Medical Science.

Note that full information on the approval of the study protocol must also be provided in the manuscript.

Flow Cytometry

Plots

Confirm that:

☒ The axis labels state the marker and fluorochrome used (e.g. CD4-FITC).

☒ The axis scales are clearly visible. Include numbers along axes only for bottom left plot of group (a 'group' is an analysis of identical markers).

☐ All plots are contour plots with outliers or pseudocolor plots.

☐ A numerical value for number of cells or percentage (with statistics) is provided.

Methodology

Sample preparation

Thymus were isolated from non-diabetic and diabetic mice perfused with PBS(-) after anesthesia to prepare the suspensions of the thymocyte. Thymocyte suspensions were stained with LIVE/DEAD violet dead cell stain kit (ThermoFisher Scientific) for 30 minutes to remove dead cells, washed with PBS(-), and reacted with anti-CD16/32 antibody (Clone 93, Biolegend) to block Fc receptor. Cell suspensions were then stained with PECy7 CD3 (Clone 17A2, Biolegend), FITC CD4 (Clone GK1.5, Biolegend), and APC CD8a (Clone 53-6.7, Biolegend) antibodies for 30 minutes. The suspensions of stained cell were analyzed using FACS Aria Fusion (BD bioscience).

Instrument

FACSAria Fusion (BD Biosciecnes)

Software

FACS Diva4.0 software (BD Biosciences)

Cell population abundance

Describe the abundance of the relevant cell populations within post-sort fractions, providing details on the purity of the samples and how it was determined.

Gating strategy

A portion of unstained suspended cells was stained with an isotype control of each fluorescent antibody to determine gating. Acquired data were analyzed using FACS DIVA software (BD Bioscience).

☐ Tick this box to confirm that a figure exemplifying the gating strategy is provided in the Supplementary Information.
